# Effect of outdoor activity on myopia onset and progression in school-aged children in northeast china: the sujiatun eye care study

**DOI:** 10.1186/s12886-015-0052-9

**Published:** 2015-07-09

**Authors:** Ju-Xiang Jin, Wen-Juan Hua, Xuan Jiang, Xiao-Yan Wu, Ji-Wen Yang, Guo-Peng Gao, Yun Fang, Chen-Lu Pei, Song Wang, Jie-Zheng Zhang, Li-Ming Tao, Fang-Biao Tao

**Affiliations:** Department of Maternal, Child and Adolescent Health, School of Public Health, Anhui Medical University, 81st Meishan Road, Hefei, 230032 Anhui Province P.R China; Health Center for Elementary and Middle School Students of Sujiatun District, Shenyang, Liaoning China; Shenyang Aier Eye Hospital, Shenyang, Liaoning China; Department of Ophthalmology, the Second Hospital of Anhui Medical University, Hefei, Anhui China; Education Bureau of Sujiatun District, Shenyang, Liaoning China; Anhui Provincial Key Laboratory of Population Health & Aristogenics, Hefei, Anhui China

**Keywords:** Myopia, Refractive error, Outdoor activity, Schoolchildren, Intervention studies

## Abstract

**Background:**

Due to its high prevalence and associated sight-threatening pathologies, myopia has emerged as a major health issue in East Asia. The purpose was to test the impact on myopia development of a school-based intervention program aimed at increasing the time student spent outdoors.

**Methods:**

A total of 3051 students of two primary (grades 1-5, aged 6-11) and two junior high schools (grades 7-8, aged 12-14) in both urban and rural Northeast China were enrolled. The intervention group (n = 1735) unlike the control group (n = 1316) was allowed two additional 20-min recess programs outside the classroom. A detailed questionnaire was administered to parents and children. Uncorrected visual acuity (UCVA) was measured using an E Standard Logarithm Vision Acuity Chart (GB11533-2011) at baseline, 6-month and 1-year intervals. A random subsample (n = 391) participated in the clinic visits and underwent cycloplegia at the beginning and after 1 year.

**Results:**

The mean UCVA for the entire intervention group was significantly better than the entire control group after 1 year (P < 0.001). In the subgroup study, new onset of myopia and changes in refractive error towards myopia were direction during the study period was significantly lower in the intervention group than in the control group (3.70 % vs. 8.50 %, P = 0.048; -0.10 ± 0.65 D/year vs. -0.27 ± 0.52 D/year, P = 0.005). Changes in axial length and IOP were also significantly lower following the intervention group (0.16 ± 0.30 mm/year vs. 0.21 ± 0.21 mm/year, P = 0.034; -0.05 ± 2.78 mmHg/year vs. 0.67 ± 2.21 mmHg/year, P = 0.006).

**Conclusions:**

Increasing outdoor activities prevented myopia onset and development, as well as axial growth and elevated IOP in children.

**Trial registration:**

Current controlled trials NCT02271373.

**Electronic supplementary material:**

The online version of this article (doi:10.1186/s12886-015-0052-9) contains supplementary material, which is available to authorized users.

## Background

China has one of the world’s highest myopia rates with an estimated prevalence of 9.7 % in 7-year-old children, 43.8 % in 12-year-old children, and 72.8 % in 18-year-old teenagers [1]. The prevalence of myopia in rural areas is slightly lower [2–4]. In addition, the prevalence of high myopia (over -6.0D) increased from 10.9 % in 1983 to 21 % of 18-year-old students of Taiwan in 2000 [5]. There has been a tendency of myopia towards higher prevalence, greater severity (prevalence of high myopia) and younger age of onset [5–8]. Studies have shown that myopia develops rapidly in children at a younger age [9, 10]. Moreover, younger age at first diagnosis is a significant risk factor for high myopia in adult life [11–13]. High myopia is a public health and economic challenge due to devastating visual prognosis associated with complications such as glaucoma, myopic retinopathy, and retinal detachment [8, 14]. Therefore, it is imperative to develop effective interventions to prevent myopia onset and progression as much as possible.

For several decades, environmental factors have been believed to play an important role in the determination of refractive error [6, 7]. Even though a substantial proportion of myopia cases can be explained by inheritance [15], it does not exclude strong environmental etiology, especially in East Asia [16]. The past decade has witnessed a large increase in the number of observational studies investigating the hypothesis that time spent outdoors protects against myopia.

Compared with a 28 % myopia rate among Singaporean Chinese youth, the prevalence rate among Chinese youth of similar age living in Sydney (Australia) is only 3.3 %. The main factor driving the disparity is attributed to the difference in time spent outdoors, estimated at 13.8 h per week in Sydney compared with 3.0 h per week in Singapore [17]. A prospective cohort study showed that children with myopia spent significantly fewer hours per week in outdoor/sports activities compared with emmetropes before, during, and after myopia onset [18]. Guggenheim et al. [19] found that time spent outdoors was predictive of incident myopia independently of the physical activity level. As documented in a recent meta-analysis, each increase in hours per week spent outdoors was associated with a 2 % reduced odds of myopia, after adjustment for covariates [20].

In addition, data from interventional studies showed that outdoor activity during class recess or an additional class of structured outdoors activities after school led to a significant effect on myopia onset and myopic shift [21, 22]. Furthermore, evidence of animal studies suggested that the development of form deprivation myopia (FDM) was reduced if diffusers were removed for a period of 15 min per day under normal laboratory light levels [23], and the protective effect was enhanced by exposure to light during the diffuser-free period, proportional to the light intensity used [24].

With the hypothesis that increasing time spent outdoors may be beneficial for the visual health of children, we included two extra 20-min recess programs five days per week among school-aged children in northeast China. In our study, we compared myopia onset and progression and ocular biometric parameters between intervention and control arms.

## Methods

### Participants

The Sujiatun Eye Care Study was a school-based, prospective, interventional study performed in a representative county (Sujiatun District, Shenyang) of northeast China. There were 25 primary schools and 19 junior high schools, and about 25,000 children in these schools. Students of two primary (grades 1-5, aged 6-11) and two junior high schools (grades 7-8, aged 12-14) in both urban and rural locations participated. Students of grades 6 and 9 progressed to higher levels in September the following year, and were excluded in our study to avoid loss of follow-up. Two nearby primary schools in urban areas with comparable academic level were assigned to the intervention arm or control arm randomly. The two urban junior high schools, two rural primary schools and two rural junior high schools were assigned similarly. The study protocol was approved by the Human Research Ethics Committee of Anhui Medical University and adhered to the Declaration of Helsinki. After explaining the nature of the study to children and parents, written informed consent was obtained from at least 1 parent, and verbal agreement to participate in the study had been obtained from the children.

### Interventions

The interventions included two additional recess programs of 20 minutes each outside the classroom that encouraged children to venture into outdoor activities during recess in the morning and afternoon during school days within a period of 1 school year. In the intervention schools, at 9:30 a.m. the original 10-minute break was stretched to 30 minutes and similarly at 2:30 p.m. The school hours was extended by 40 minutes. It was compulsory for students to go outside during recess, and the teacher in charge was responsible for ensuring that students participate in outdoor activities during recess. The two recess programs were included in the daily schedule for students through the Education Bureau of Sujiatun District. In addition, we provided students with free rope skipping, shuttlecock, badminton and other equipments. No interventions were included in the control school.

### Questionnaire

All students and their parents completed a detailed questionnaire (The Sujiatun Eye Care Study Questionnaire, Additional file 1, available at http://www.biomedcentral.com/imedia/5773752031744030/supp1.doc) including children’s gender, ethnicity, region of habitation; parental education level, the monthly family income and parents’ myopic status. The questionnaire also included items related to the amount of time spent in learning (reading or writing), screen time (television, computer, etc) and time spent outdoors after school in recent 7 days. The average number of daily activity (learning, screen time, and outdoor activity) hours was calculated using the following formula: [(hours spent on a weekday) × 5+ (hours spent on a weekend day) × 2]/7.

### School-based ametropia screening

All students in the selected intervention and control schools undertook the school-based ametropia screening which was conducted by a clinical team of 4 groups; each group consisting of 2 optometrists from Shenyang Aier Ophthalmology Hospital, assisted by 1 post-graduate student from the School of Public Health, Anhui Medical University at baseline, at 6 months and at 1 year. School doctors and class teachers were asked to maintain order during school-based ametropia screening. Uncorrected visual acuity (UCVA) was measured for the right eye, followed by the left eye, with an E Standard Logarithm Vision Acuity Chart (GB11533-2011) in 5-grade notation, with illumination of the chart around 500 lx [25]. The 5-grade notation was obtained using the formula: L = 5- LogMAR. The child was asked to indicate the direction of the E optotype within 5 seconds. Measurements commenced at a distance of 5 m, with the 5.0 line optotypes of the chart and the eyes of the tested children approximately at equal height. The children were asked to start with the fourth line from the bottom (5.0), and proceed to the next line, if optotypes of the line were correctly described, otherwise, they continued with the previous line. When the children falsely described at least 1 character in the 5.1 to 5.3 line, or at least 2 characters in the 4.6 to 5.0 line, or at least 3 characters in the 4.0 to 4.5 line, visual acuity was recorded as the value of the previous line. In China, a score of 5.0 in 5-grade notation, equaling 1.0 in decimal notation, is defined as standard vision. If the UCVA was less than 5.0, tests with a combination of lenses (a series of concave lenses and convex lenses) were conducted to confirm the type of ametropia (normal, suspected myopia, suspected hyperopia or suspected other eye diseases). VA examinations were carried out using a uniform protocol throughout the study periods. All the measurements were recorded.

### Subgroup study

A random subsample (about 12.8 %) participated in the clinic visits and underwent cycloplegia at baseline and intervention for 1 year. Cycloplegia of all students was precluded by high equipment costs. After explaining the side effects of cycloplegia to children and parents, written informed consent was obtained from at least 1 parent, and verbal agreement to participate in the study obtained from the children. After excluding the risk of a medical mydriasis, cycloplegia was performed at baseline and intervention after 1 year in the Shenyang Aier Ophthalmology Hospital, respectively. Ocular biometric parameters (axial length, corneal curvature, anterior chamber depth and IOP) were measured in both eyes of all participants. Axial length, corneal curvature and anterior chamber depth were measured with IOL Master (Carl Zeiss Meditex, Jena, Germany); IOP was measured using non-contact tonometry (NT-510, NIDEK, Gamagori, Japan). Cycloplegia was achieved with 0.5 % tropicamide eye drops. In total six drops of tropicamide 0.5 % were administered at 5-minute intervals to both eyes. Refractometry was performed 20 minutes after the last cycle of cycloplegic eye drops by retinoscopy (YZ-24, Suzhou, China). All examinations were conducted by trained ophthalmologists and optometrists and the same apparatus was used in 2012 and 2013. The spherical equivalent of the refractive error (SER) was calculated as spherical refractive error + 1/2 cylindrical refractive error. Myopia was defined as refractive error (spherical equivalent) of ≤ -0.50D in the right eye.

### Statistical analysis

We used data from the right eye only, based on the generally high correlation between the left and right eyes. UCVA in 5-grade notation was transformed into logMAR notation in the statistical analysis. The progression of myopia was calculated as the change in SER during 1 year. Quantitative observations were presented as mean ± SD. Student’s t-tests were used to compare quantitative data between analyzed variables and myopia. Categorical data were compared by chi-square tests. Differences in the three UCVA results between the two groups were analyzed using multivariate analysis of variance for repeated measures. All statistical analyses were performed using the Statistical Package of Social Sciences and Problem Solutions (SPSS, version 13.0; SPSS, Inc., Chicago, IL). P < 0.05 was considered statistically significant.

## Results

**Fig. 1:**
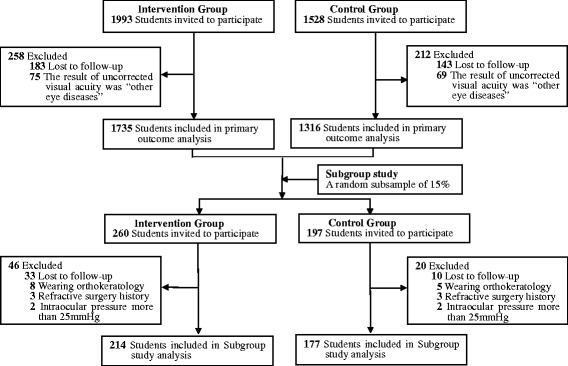
Flowchart Detailing Sample Selecting

Figure 1 displays the flow diagram of determining the eligibility of participants for the final analysis.

The 1-year intervention was conducted between November 2012 and November 2013.

**Fig. 2:**
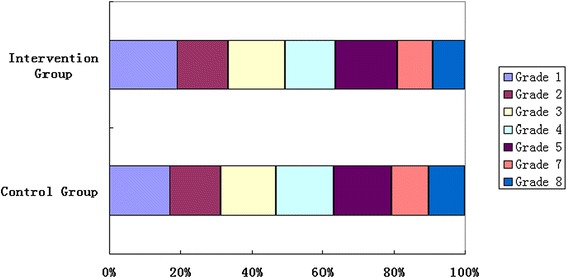
Bar graph showing the ratio of students of each grade in control group and intervention group. The two groups were comparable (*χ* ^*2*^ = 5.45, P = 0.487)

Out of 3521 eligible students in both intervention and control groups, only 3051 (86.7 %) children were included in the study. Among the 470 children excluded, 326 were lost to follow-up and 144 children were found with “suspected other eye diseases” following ametropia screening (Fig. 1). Hence, statistical analyses were based on data from 3051 (86.7 %) students. Among the study participants, 1539 (50.4 %) were living in the rural region, and 1512 (49.6 %) were urban; 2451 (80.3 %) children attended the primary school, and 600 (19.7 %) went to the junior high school. No statistical differences were found in gender, age and region of habitation between participants and non-participants. Table 1 presents summary statistics for the study groups at baseline. There were no significant differences in the two groups in terms of gender, age, nationality, region of habitation, baseline UCVA, prevalence of suspected myopia, daily screen time (television, computer, etc), daily study duration (reading or writing) and daily outdoor activity time. Further, the ratio of students of each grade in the control and intervention groups was also comparable (*χ*^*2*^ = 5.45, P = 0.487; Fig. 2). However, when compared with control group, the intervention group had more myopic parents, higher parental education and higher individual income.

**Table 1:** Baseline profile of participants

Characteristics	Intervention group	Control group	*P* Value
	(*n* = 1735)	(*n* = 1316)	
Gender (%)			0.297
Male	874(50.4)	688(52.3)	
Female	861(49.6)	628(47.7)	
Mean age ± SD (yrs)	10.09 ± 2.35	10.25 ± 2.33	0.060
Nationality (%)			0.272
Han nationality	1459(84.1)	1087(82.6)	
Others	276(15.9)	229(17.4)	
Region of habitation (%)			0.373
Rural	863(49.7)	676(51.4)	
Urban	872(50.3)	640(48.6)	
Mean baseline UCVA ± SD	4.88 ± 0.20	4.88 ± 0.22	0.713
Suspected myopia (%)			0.195
Yes	410(31.2)	579(33.4)	
No	906(68.8)	1156(66.6)	
Myopic parents(%)			<0.001
Yes	525(30.3)	318(24.2)	
No	1210(69.7)	998(75.8)	
Paternal education(%)			< 0.001
Primary school or less	194(11.2)	154(11.7)	
Junior middle school	788(45.4)	724(55.0)	
Senior middle school	472(27.2)	327(24.8)	
College or above	281(16.2)	111(8.4)	
Maternal education(%)			< 0.001
Primary school or less	225(13.0)	226(17.2)	
Junior middle school	826(47.6)	702(53.3)	
Senior middle school	432(24.9)	276(21.0)	
College or above	252(14.5)	112(8.5)	
Family income per person (%)			< 0.001
< 2000 RMB	268(15.4)	264(20.1)	
2000-3999RMB	646(37.2)	592(45.0)	
4000-5999RMB	534(30.8)	358(27.2)	
6000-9999 RMB	238(13.7)	88(6.7)	
10000+ RMB	49(2.8)	14(1.1)	
Daily screen time*	1.94 ± 1.70	1.83 ± 1.43	0.067
Daily study duration*	1.50 ± 1.25	1.59 ± 1.50	0.058
Daily outdoor activity time*	1.20 ± 1.69	1.18 ± 1.55	0.723

*UCVA* uncorrected visual acuity

*Data are expressed as the mean ± SD

**Fig. 3:**
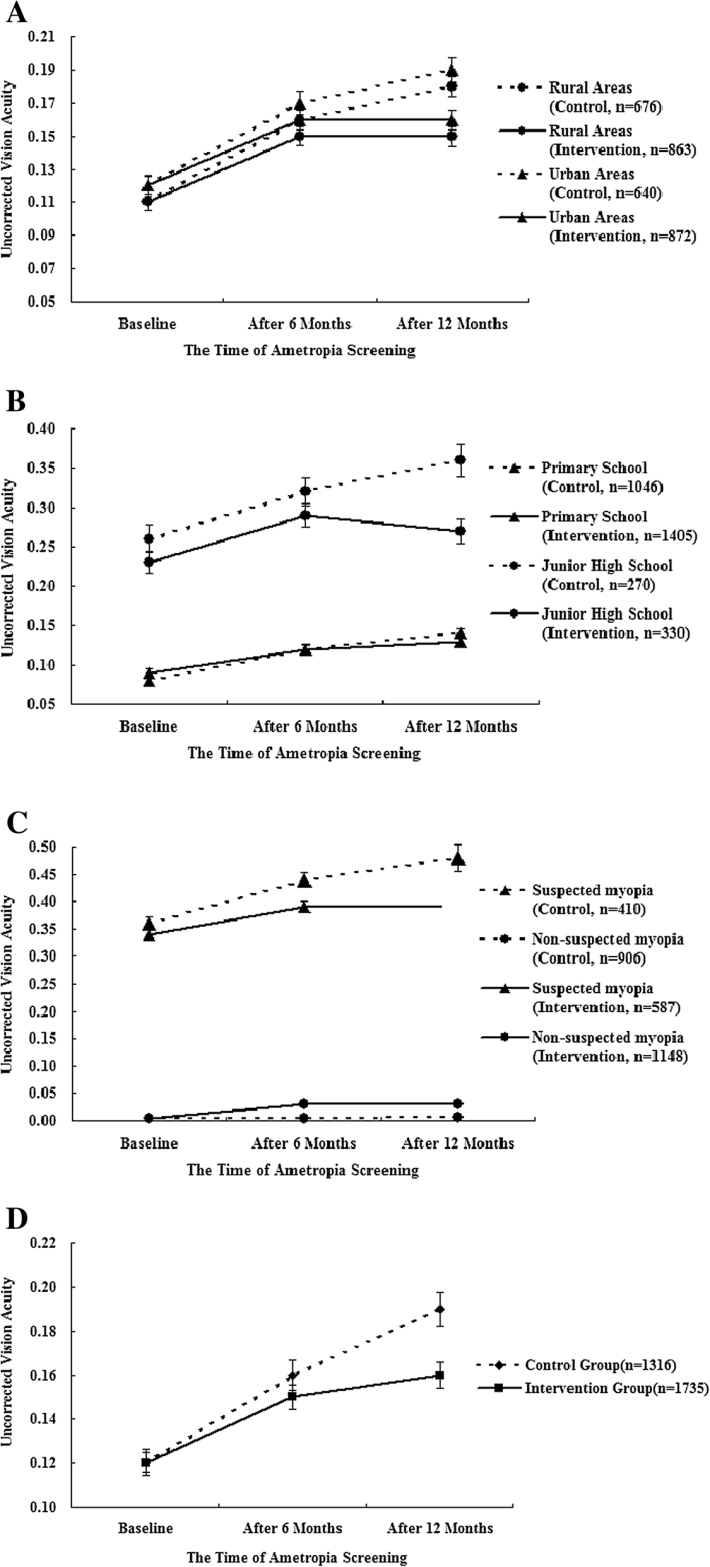
**a** Comparison of mean uncorrected visual acuity (logMAR notation) between groups by different locations (rural areas vs. urban areas). Multivariate analysis of variance of mean uncorrected visual acuity during the 1-year follow-up period showed statistical significance (*P* < 0.001). **b** Comparison of mean uncorrected visual acuity (logMAR notation) between groups by different grade levels (primary school vs. junior high school). Multivariate analysis of variance of mean uncorrected visual acuity during the 1-year follow-up period showed statistical significance (*P* < 0.001). **c** Comparison of mean uncorrected visual acuity (logMAR notation) between groups by different visual acuity at baseline (suspected myopia or not). Multivariate analysis of variance of mean uncorrected visual acuity during the 1-year follow-up period showed statistical significance (*P* < 0.001). **d** Comparison of mean uncorrected visual acuity (logMAR notation) between groups. Multivariate analysis of variance of mean uncorrected visual acuity during the 1-year follow-up period showed statistical significance (*P* < 0.001)

The mean UCVA measurements of the intervention and control groups are listed in Table 2. The UCVA changed significantly after 1-year intervention in different regions of habitation (both P < 0.001, Fig. 3a), and with different grade levels (both P < 0.001, Fig. 3b) for both group. There was also a significant change in suspected myopia and non-suspected myopia in both groups after 1-year intervention (both P < 0.001, Fig. 3c). All the participants in each of the intervention and control groups exhibited similar results (Fig. 3d).

**Table 2:** Comparison of uncorrected visual acuity (LogMAR notation) between groups

	Intervention group			Control group			*P* Value^*^
	T1^a^	T2^a^	T3^a^	T1^a^	T2^a^	T3^a^	
Rural (n = 1539)	0.11 ± 0.20	0.15 ± 0.23	0.15 ± 0.24	0.11 ± 0.20	0.16 ± 0.23	0.18 ± 0.24	< 0.001
Urban (n = 1512)	0.12 ± 0.21	0.16 ± 0.23	0.16 ± 0.23	0.12 ± 0.24	0.17 ± 0.27	0.19 ± 0.29	< 0.001
Elementary school (n = 2451)	0.09 ± 0.18	0.12 ± 0.21	0.13 ± 0.22	0.08 ± 0.18	0.12 ± 0.22	0.14 ± 0.22	< 0.001
Secondary school (n = 600)	0.23 ± 0.26	0.29 ± 0.28	0.27 ± 0.28	0.26 ± 0.29	0.32 ± 0.30	0.36 ± 0.34	< 0.001
Suspected myopia (n = 997)	0.34 ± 0.20	0.39 ± 0.24	0.39 ± 0.24	0.36 ± 0.24	0.44 ± 0.27	0.48 ± 0.29	< 0.001
Non-suspected myopia (n = 2054)	0.005 ± 0.05	0.03 ± 0.09	0.03 ± 0.09	0.004 ± 0.04	0.039 ± 0.09	0.06 ± 0.10	< 0.001
Combined (n = 3051)	0.12 ± 0.20	0.15 ± 0.23	0.16 ± 0.24	0.12 ± 0.22	0.16 ± 0.25	0.19 ± 0.27	< 0.001

T1 means baseline; T2 means after 6 months; T3 means after 12 months

^a^Data are expressed as the mean ± SD

^*^Multivariate analysis of variance for repeated measures between groups

### Subgroup study

We selected 457 students (about 15 % of the whole sample) randomly using stratified-cluster sampling from 16 classes in grade 1, grade 3, grade 5 and grade 7 for the subgroup study. Finally, 391 (about 12.8 %) children with complete data underwent cycloplegia. Among the 66 children excluded, 43 were lost to follow-up, 13 were wearing orthokeratology lenses, 6 had a history of refractive surgery and the IOP in 4 children exceeded 25 mmHg (Fig. 1). There were no statistical differences in gender, age, region of habitation and baseline UCVA between subgroup participants and non-participants. Finally, there were 214 children in the intervention group and 177 in the control group (Table 3). The control and intervention groups were fairly comparable with no statistical differences in gender, age, nationality, region of habitation and baseline SER (P = 0.184, P = 0.159, P = 0.352, P = 0.46, P = 0.825). Based on the response to the questionnaire, the baseline estimated time spent for learning (reading or writing), screen time (television, computer, etc) and time spent outdoors after school was also not significantly different between groups (P = 0.179, P = 0.991 and P = 0.164, respectively). However, when compared with control group, the intervention group had more myopic parents, higher parental education level and higher individual income.

**Table 3:** Baseline comparison of participants in the subgroup study

Characteristics	Intervention group (*n* = 214)	Control group (*n* = 177)	P Value
Gender (%)			0.184
Male	116(54.2)	84(47.5)	
Female	98(45.8)	93(52.5)	
Mean age ± SD (yrs)	10.77 ± 2.14	10.42 ± 2.72	0.159
Nationality (%)			0.352
Han nationality	183(85.5)	157(88.7)	
Others	31(14.5)	20(11.3)	
Region of habitation (%)			0.460
Rural	102(47.7)	91(51.4)	
Urban	112(52.3)	86(48.6)	
Mean baseline SER ± SD	−0.83 ± 1.54	−0.87 ± 1.68	0.825
Myopic parents(%)			0.023
Yes	72(33.6)	41(23.2)	
No	142(66.4)	136(76.8)	
Paternal education(%)			0.042
Primary school or less	24(11.2)	31(17.5)	
Junior middle school	121(56.5)	108(61.0)	
Senior middle school	52(24.3)	32(18.1)	
College or above	17(7.9)	6(3.4)	
Maternal education(%)			0.001
Primary school or less	27(12.6)	37(20.9)	
Junior middle school	116(54.2)	110(62.1)	
Senior middle school	56(26.2)	28(15.8)	
College or above	15(7.0)	2(1.1)	
Family income per person (%)			0.004
< 2000 RMB	62(29.0)	71(40.1)	
2000-3999RMB	106(49.5)	91(51.4)	
4000-5999RMB	33(15.4)	13(7.3)	
6000-9999 RMB	9(4.2)	1(0.6)	
10000+ RMB	4(1.9)	1(0.6)	
Daily screen time*	1.75 ± 0.83	1.75 ± 0.81	0.991
Daily study duration*	1.55 ± 0.95	1.67 ± 0.88	0.179
Daily outdoor activity time*	1.64 ± 0.97	1.78 ± 0.89	0.164

*Data are expressed as the mean ± SD

At the end of the 1-year follow-up, the incidence of new myopia onset during the study period was lower in the intervention group than in the control group (8 students vs. 15 students; 3.70 % vs. 8.50 %, P = 0.048). In addition, the SERs at the final examination were -0.93 ± 1.50 D in the intervention group and -1.13 ± 1.67 D in the control group (P = 0.202). The mean progression of refractive error in the myopic direction was significantly lower in the intervention group than in the control group (-0.10 ± 0.65 D/year vs. -0.27 ± 0.52 D/year, P = 0.005). In addition, changes in axial length and IOP were also significantly lower in the intervention group than in the control group (0.16 ± 0.30 mm/year vs. 0.21 ± 0.21 mm/year, P = 0.034; -0.05 ± 2.78 mmHg/year vs. 0.67 ± 2.21 mmHg/year, P = 0.006). Corneal curvature and anterior chamber depth changes showed no significant differences between groups (Table 4).

**Table 4:** Ocular biometric analysis in subgroup study

Parameter	Intervention group (n = 214)			Control group (n = 177)		
	Initial	Final	Change	Initial	Final	Change
SER(D)	−0.83 ± 1.54	−0.93 ± 1.50	−0.10 ± 0.65**	−0.87 ± 1.68	−1.13 ± 1.67	−0.27 ± 0.52
AL (mm)	23.85 ± 0.98	24.01 ± 1.01	0.16 ± 0.30*	23.68 ± 0.91	23.89 ± 0.97	0.21 ± 0.21
Corneal curvature (D)	43.15 ± 1.50	43.26 ± 1.50	0.11 ± 0.31	43.41 ± 1.47	43.50 ± 1.48	0.09 ± 0.48
Anterior chamber depth (mm)	3.59 ± 0.26	3.63 ± 0.24	0.04 ± 0.17	3.54 ± 0.23	3.59 ± 0.24	0.04 ± 0.09
IOP (mmHg)	16.63 ± 2.75	16.57 ± 2.93	−0.05 ± 2.78**	16.21 ± 2.55	16.88 ± 2.63	0.67 ± 2.21

*SER* spherical equivalent refraction; *AL* axial length; *D* diopters; *IOP* intraocular pressure

*compared with the control group, *P* < 0.05

** compared with the control group, *P* < 0.01

## Discussion

Our study findings support the role of outdoor activities in the prevention of myopia onset and myopic shift among primary and junior high school students in northeast China. Increasing time spent outdoors also had a significant effect on axial length elongation and elevated IOP. However, our study found no relationship between the intervention program and changes in corneal curvature and anterior chamber depth, probably due to the small sample size of the subgroup, resulting in low statistical power.

Prior to our intervention, most of the students stayed in the classroom during recess time for indoor work. After intervention, it was compulsory for students to go outside during the two 20-min breaks, and the teacher in charge was responsible for organizing the outdoor activities during recess. Thus, the two recess programs interrupted indoor work during the classroom hours, effectively. A school-based cross-sectional study showed that more indoor study was associated with myopia in grade 1 and grade 4 primary school children in Greater Beijing [4]. The study by Ip JM et al. [26] showed that continued reading is associated with myopia. The intensity rather than the total duration of near work is a key factor. Our recess program provided a break from continued near-range work and reduced its intensity. Furthermore, the two recess programs increased the time spent outdoors for school children. Recent studies suggest that outdoor activity was an important protective factor against myopia [27]. In addition, the COMET group estimated 15.61 ± 4.17 years as the age of myopia at stabilization in an ethnically diverse cohort [28]. Our study participants were younger than their estimated age, which may have resulted in effective outcomes following the intervention.

In the whole sample, the speed of UCVA loss was slower in the intervention group than the control group at 6 months, and differences between groups reached statistical significance at 12 months. Our results confirmed the previous studies showing that outdoor activity might protect against development of myopia in children [4, 29, 30]. A recent prospective interventional study of children aged 7-11 years [21] showed that locking classroom doors during school recess, which prevented the children from staying indoors and working, had a significant effect on myopia onset (8.41 % vs. 17.65 %; P < 0.001) and myopic shift (-0.25 ± 0.68D/year vs. -0.38 ± 0.69 D/year; P = 0.029), consistent with our subgroup study findings (3.70 % vs. 8.50 %, P = 0.048 for myopia onset; -0.10 ± 0.65 D/year vs. -0.27 ± 0.52 D/year, P = 0.005 for myopic shift). Our results are also consistent with the findings of Morgan *et al.* [22], who observed that an additional class after school including structured outdoor activities was related to a 25 % reduction in new cases of myopia compared with about 50 % reduction in new cases of myopia in this study.

Although an association between myopia prevalence and time spent outdoors was relatively consistent among several studies, the underlying mechanism of protection against myopia onset and progression was less clear. Bright light outdoors may be the most possible mechanism. Brighter light potentially reduces the development of myopia by pupil constriction, resulting in less visual blur, or by stimulation of dopamine release from the retina. Dopamine has been known to be an inhibitor of axial elongation [31]. Evidence of animal studies suggested that bright light prevented the development of myopia and the protective effect was blocked by the dopamine antagonist spiperone [32, 33]. The results of these experimental studies support the results of Rose *et al.* [17, 29], Guo *et al.* [4], and other studies, as well as our results since outdoor activity is associated with the exposure to bright light. It may also be hypothesized that biochemical changes related to increased physical activity inhibited the development of eye disorders.

Notably, the results of our study showed that increasing time spent outdoors via recess programs might retard axial length elongation and elevated IOP in school-aged children. Compared with other ocular components such as the cornea, crystalline lens and vitreous body, axial length is regarded as the primary determinant of refractive error. According to a multiple linear regression analysis refractive error was associated with axial length more than any other parameter [34]. The correlation between change in axial length and progression of myopia, documented in randomized clinical trials, is also quite high, ranging between 0.77 and 0.89 [35, 36]. The CLEERE (Collaborative Longitudinal Evaluation of Ethnicity and Refractive Error) study demonstrated that myopic children had longer axial lengths than did emmetropes before and after the onset of myopia (3 years before through 5 years after), with the fastest rate of change in axial length occurring during the year before rather after onset [37]. Axial length and associated variation may therefore, be potentially useful in predicting the onset of myopia. Slowing down the axial growth was of great significance in preventing myopia development. This study revealed that the rate of change in axial length was significantly lower in the intervention group when compared with the controls (0.16 ± 0.30 mm/year vs. 0.21 ± 0.21 mm/year; P = 0.034). The school-based Guangzhou Outdoor Activity Longitudinal (GOAL) Study on children aged 6 to 7 years found that children who had one extra hour of structured outdoor activities added to the school day had statistically significant reductions in axial elongation (0.29 ± 0.18 mm/year vs. 0.33 ± 0.23 mm/year) after 1 year [22]. The rate of change in axial length was slower in our Shenyang study population than in the GOAL study population. Most of the discrepancy was associated with the much older age in our participants (6 to 14 years vs. 6 to 7 years).

The mean IOP of our study was 16.44 ± 2.67 mmHg, which was similar to a previous report of the Beijing eye study (16.11 ± 3.39 mmHg) [38]. However, it is higher than those of previous reports from other Asian countries (14.6 ± 2.7 mmHg in Tajimi study; 13.99 ± 2.75 mmHg in the Korea National Health and Nutrition Examination Survey) [39, 40]. The explanation for our higher IOP is unclear, although presumably related to the specific population differences, different range of age or other unknown factors. A few previous studies have reported that IOP was positively associated with the degree of myopia or axial elongation [41–43]. However, it was not consistent with findings from the Los Angeles Latino Eye Study who failed to observe the link between IOP and axial length [44]. Our study found that the rate of change in IOP was significantly lower in the intervention group when compared with the controls (-0.05 ± 2.78 mmHg/year vs. 0.67 ± 2.21 mmHg/year, P = 0.006). However, the differences are not clinically significant. Out results might be a valuable reference for researchers in the future studies.

The limitations of this study include the lack of individual randomization, and the lack of precise hours in outdoor lighting for each individual. It is realistic to randomize the children in each school, but a contamination effect cannot be ruled out, for example, children in the intervention group might associate with their friends from the control group in the outdoor activies during recess. In addition, the intervention and control groups were not entirely comparable at baseline. As compared with control group, the intervention group had more myopic parents, higher parental education level and higher individual income. However, previous researches suggested that more myopic parents, higher parental education level and higher individual income were risk factors for childhood myopia. Despite these risk factors in the intervention group, the intervention with increased time spent outdoors still showed significant preventive effect against myopia.

## Conclusions

In summary, the current findings suggested that increasing time spent outdoors by conducting recess program leads to a significant control of UCVA loss, and myopia onset and progression among school-aged children in northeast China. It is expected that the results will provide evidence for policy-makers and school healthcare providers for myopia prevention. The repeatability of these findings and the biological significance of these variations with respect to myopia have yet to be confirmed in additional large randomized controlled trials using similar or new outdoor interventions in schools to prevent and evaluate myopic onset and progression.

## Additional file

Additional file 1:
**The Sujiatun Eye Care Study Questionnaire.**

## Abbreviations

UCVA: Uncorrected visual acuity
IOP: Intraocular pressure
SER: Spherical equivalent of the refractive error
SD: Standard deviation
D: Diopters
AL: Axial length
