# Supplementary material for: Effect of outdoor activity on myopia onset and progression in school-aged children in northeast china: the sujiatun eye care study
Source: BMC Ophthalmol. 2015 Jul 9;15:73. doi: 10.1186/s12886-015-0052-9 (PMC4495846; doi:10.1186/s12886-015-0052-9)
Supplement: Additional file 1: — The Sujiatun Eye Care Study Questionnaire. [file 12886_2015_52_MOESM1_ESM.doc]

**用眼行为调查问卷**

**一、学生情况**

| **姓名** | | **学 校** | | |
| --- | --- | --- | --- | --- |
| **年级** | | | | **班级** |
| **性别** ①男 ②女 | **民族** ①汉族 ②其他 | | | |
| **家庭所在地** ①农村 ②城市 | | | | |
| **出生日期（阳历）** 年 月 日 | | | **填表日期**  年 月 日 | |
| **身高 .** 厘米 | | | **体重 . 斤** | |

**二、父母情况**

**1. 生父出生日期（阳历）** 年 月 日;

**2. 生母出生日期（阳历）** 年 月 日;

**3. 生父学历**

①小学及以下 ②初中 ③高中 ④大专及以上

**4. 生母学历**

①小学及以下 ②初中 ③高中 ④大专及以上

**5. 生父是否近视** ①是 ②否

**6. 生母是否近视**  ①是 ②否

**7. 你家每月的家庭收入约为多少元？**

①2000元以下 ②2000～3999元 ③4000～5999元 ④6000～9999元 ⑤10 000元以上

**三、请你仔细回忆一下，最近7天你的用眼行为。**

| 上学日（周一至周五）读书或写字时间 | 平均每天 分钟 |
| --- | --- |
| 上学日（周一至周五）看电视时间 | 平均每天 分钟 |
| 上学日（周一至周五）使用电脑时间 | 平均每天 分钟 |
| 上学日（周一至周五）玩手机时间 | 平均每天 分钟 |
| 周末读书或写字时间 | 平均每天 分钟 |
| 周末看电视时间 | 平均每天 分钟 |
| 周末使用电脑时间 | 平均每天 分钟 |
| 周末玩手机时间 | 平均每天 分钟 |

**四、体育活动**

**1.** 请您仔细回忆一下，**最近3个月以来，**你是否参加过下列活动。若有，请在相应的选项上打“√”，并估计你平均**每周**参加该项活动的次数及每次参加活动的时间。

| **活动类型** | **是否参加过** | **平均每周参加该活动的次数** | **每次活动时间** | **参加该活动的场所** |
| --- | --- | --- | --- | --- |
| 舞蹈或体操（含健美操） | ①Yes ②No | 次/每周 | 小时/每次 | ①室内 ②室外 |
| 快走或跑步 | ①是 ②否 | 次/每周 | 小时/每次 | ①室内 ②室外 |
| 游泳 | ①是 ②否 | 次/每周 | 小时/每次 | ①室内 ②室外 |
| 足球 | ①是 ②否 | 次/每周 | 小时/每次 | ①室内 ②室外 |
| 羽毛球 | ①是 ②否 | 次/每周 | 小时/每次 | ①室内 ②室外 |
| 乒乓球 | ①是 ②否 | 次/每周 | 小时/每次 | ①室内 ②室外 |
| 篮球 | ①是 ②否 | 次/每周 | 小时/每次 | ①室内 ②室外 |
| 滑板/轮滑/滑冰 | ①是 ②否 | 次/每周 | 小时/每次 | ①室内 ②室外 |
| 跳绳/跳皮筋/踢毽子 | ①是 ②否 | 次/每周 | 小时/每次 | ①室内 ②室外 |
| 扔沙包、铅球等投掷运动 | ①是 ②否 | 次/每周 | 小时/每次 | ①室内 ②室外 |
| 武术/跆拳道 | ①是 ②否 | 次/每周 | 小时/每次 | ①室内 ②室外 |
| 集体游戏 | ①是 ②否 | 次/每周 | 小时/每次 | ①室内 ②室外 |
| 骑自行车 | ①是 ②否 | 次/每周 | 小时/每次 | ①室内 ②室外 |
| 其他 | ①是 ②否 | 次/每周 | 小时/每次 | ①室内 ②室外 |

**The Sujiatun Eye Care Study Questionnaire**

**Part One**

| **Name** | | **School** | | |
| --- | --- | --- | --- | --- |
| **Grade** | | | | **Class** |
| **Gender** ① boy； ② girl | **Nationality**  ①Han nationality ②Others | | | |
| **Registered residence**  ① urban； ② rural | | | | |
| **Birth date** | | | **Date** | |
| **Height .** cm | | | **Weight . kg** | |

**Part Two**

**1. Father’s date of birth**

**2. Mother’s date of birth**

**3.** **How much education does your father have?**

① Primary school or less； ② Junior middle school；

③ Senior middle school； ④ College or above

**4.** **How much education does your mother have?**

① Primary school or less； ② Junior middle school；

③ Senior middle school； ④ College or above

**5. Does your biological father have myopia?** ① yes； ② no

**6. Does your biological mother have myopia?**  ① yes； ② no

**7. What is the monthly family income per peron?**

① < 2000 RMB ② 2000-3999RMB ③ 4000-5999RMB ④ 6000-9999 RMB ⑤10000+ RMB

**Part Three: Eye Care** Behavior

| the amount of time spent in learning (reading or writing) on a weekday | min per day in average |
| --- | --- |
| the amount of time spent in watching TV on a weekday | min per day in average |
| the amount of time spent in computer using on a weekday | min per day in average |
| the amount of time spent in mobile phone using on a weekday | min per day in average |
| the amount of time spent in learning (reading or writing) on a weekend day | min per day in average |
| the amount of time spent in watching TV on a weekend day | min per day in average |
| the amount of time spent in computer using on a weekend day | min per day in average |
| the amount of time spent in mobile phone using on a weekend day | min per day in average |

**Part Four：Activities Participating**

**Please tick the following activities which you participated in the recent 3 months, and fill the number of hours per week you spend doing the activity.**

| **Activities** | **Whether Participating** | **Average Times Per Week** | **Average Hours Every Time** | **Where is this done?** |
| --- | --- | --- | --- | --- |
| Dancing, gymnastics  martial arts | ①Yes ②No | times/per week | hours/every time | ①Indoors ②Outdoors |
| Athletics | ①Yes ②No | times/per week | hours/every time | ①Indoors ②Outdoors |
| Swimming | ①Yes ②No | times/per week | hours/every time | ①Indoors ②Outdoors |
| Football, soccer | ①Yes ②No | times/per week | hours/every time | ①Indoors ②Outdoors |
| Badminton | ①Yes ②No | times/per week | hours/every time | ①Indoors ②Outdoors |
| Table tennis | ①Yes ②No | times/per week | hours/every time | ①Indoors ②Outdoors |
| Basketball | ①Yes ②No | times/per week | hours/every time | ①Indoors ②Outdoors |
| Skating,  rollerblading | ①Yes ②No | times/per week | hours/every time | ①Indoors ②Outdoors |
| Jump rope/rubber band skipping, kicking shuttlecock | ①Yes ②No | times/per week | hours/every time | ①Indoors ②Outdoors |
| Throwing sandbags, shot put | ①Yes ②No | times/per week | hours/every time | ①Indoors ②Outdoors |
| Martial arts/tae kwon do | ①Yes ②No | times/per week | hours/every time | ①Indoors ②Outdoors |
| Group game | ①Yes ②No | times/per week | hours/every time | ①Indoors ②Outdoors |
| Ride a bike | ①Yes ②No | times/per week | hours/every time | ①Indoors ②Outdoors |
| Others | ①Yes ②No | times/per week | hours/every time | ①Indoors ②Outdoors |
